# Tenant perspectives on the implementation of the community homes for opportunity: a focused ethnographic study in Southwestern Ontario

**DOI:** 10.1186/s12889-023-15192-y

**Published:** 2023-02-08

**Authors:** Cheryl Forchuk, Sebastian Gyamfi, Heba Hassan, Bryanna Lucyk, Richard Booth

**Affiliations:** 1 Beryl and Richard Ivey Research Chair in Aging, Mental Health, Rehabilitation and Recovery, Mental Health Nursing Research Alliance, Lawson Health Research Institute, Parkwood Institute Mental Health Care Building, 550 Wellington Road, Suite B3-110, STN B, P.O. Box 5777, N6A 4V2 London, Canada; 2 Arthur Labatt School of Nursing, Western University London, London, Canada; 3 Lawson Health research institute, Parkwood Research Institute, Arthur Labatt School of Nursing, Western University, London, ON Canada; 4 Lawson Health Research Institute, Parkwood Research Institute in London, Western University, London, ON Canada

**Keywords:** CHO, HSC, Tenants, Housing, Autonomy, Recovery, Community integration

## Abstract

**Background:**

Recovery-oriented programs provide individuals with opportunities for well-being through community integration processes that enhance the degree to which individuals could live, work, and recreate in their community. The current evaluation assessed how tenants experience their home environment after the modernization of Homes for Special Care (HSC) to Community Homes for Opportunity (CHO) in Southwest Ontario, Canada. Our study identifies existing policies and practices that could interfere with or promote the modernization process.

**Methods:**

We applied ethnographic qualitative techniques to purposefully recruit 188 participants with severe mental illness from 28 group homes. Focus groups were conducted at three time points, i.e., at pre-implementation/Baseline/Time I – spring 2018; Transition/Time II – fall 2018, and Final/Time III – winter 2019.

**Results:**

Study findings suggest that the transition of HSC to CHO supports activities that empower tenants towards personal growth and development. Participants were largely satisfied with the support they were getting in relation to the program-related services. Tenants disclosed that their quality of life and well-being had been enhanced through participating in the program, and that their social interaction and support for each other had also improved. Most tenants demonstrated autonomy in terms of personal and financial independence. The enhanced financial support for tenants did not only improve their quality of life, but also helped to raise their purchasing power, decision making, sense of responsibility and accountability towards healthy spending of their resources. Despite tenants’ good impression about the CHO, some still encountered problems and provided suggestions to further improve the program.

**Conclusion:**

It is expected that a more effective and expanded CHO will lead to tenant empowerment and successful social integration.

## Background

A recovery-oriented system is one that focuses on the person by enabling individuals affected by mental illness to live independent, satisfying, hopeful, and a productive life despite limitations caused by their illness [1, 12]. Recovery-oriented programs provide individuals with opportunities for well-being through community integration processes that enhance the degree to which an individual could live, work, and recreate in their community [1, 28].

Recovery is not possible without access to housing [27]. Stable supportive housing is an essential catalyst to community integration, which then offers social connection, a feeling of belonging, and participation in the community [3, 15]. Stable housing is key to ending chronic homelessness for people living with mental illness or addictions [9, 19]. Supportive housing is guided by recovery with less emphasis on pathology and more emphasis on rehabilitation. The Supportive housing model fosters personal/individual strength building using informal and formal supports. In this model, the consumer is seen as an expert and part of shared decision-making that fosters autonomy in the end [7]. Decisions are based on the individual’s experiences and the provider’s expertise in working together [20].

In 1976, the Ministry of Health (MOH) in Ontario, Canada began providing funding to non-profit community mental health housing agencies for them to provide professional supports to individuals in addition to the custodial care they were receiving [17]. This type of group living provided with professional support by a health professional became ‘supportive housing’. In the Ontario provincial framework, supportive housing has been defined broadly as a combination of housing type, housing assistance, and supports driven by an individual’s needs for well-being [16]. Supportive housing is provided in a variety of different housing types involving group homes, clustered apartments, and halfway homes [8, 22]. In 2017, in Ontario, the Ministry of Municipal Affairs, Housing, Community and Social Services; Children and Youth Services, and the Ministry of Health formed an alliance to create a framework for modernizing supportive housing systems to a more recovery-based model. ‘The Ontario Supportive Housing Policy Framework’ included: (1) housing type, individual units that could provide either permanent or transitional supportive housing; (2) housing assistance which included rent geared-to-income, rent supplements and housing allowances; and (3) supports from case management or counselling, life skills training, and peer support [16].

Nelson and colleagues [17] state that in some supportive housing models, the level of support tenants received matched their housing type. Individuals with mental illness and/or substance use disorder had to demonstrate symptom stability or treatment compliance to live more independently. This caused individuals with improved health to be relocated to a different and more independent housing type. [3, 17]. This model was detrimental to tenants as a move to more independent housing separated them from their support network. It also robbed tenants of their choice of where and who they lived with. This graduated system of housing based on improved pathology is no longer accepted based on evidence-based practice. Today, individuals are living in ‘supportive’ or ‘supported’ housing without these conditions being placed on them [3, 17].

‘Supported’ housing is a concept from the field of developmental and physical disabilities promoted by Paul Carling and colleagues [2]. Unlike supportive housing, individuals living in supported housing are not socially integrated by their disability but by their choice of housing and the services they want. Having a choice of where to live and community services needed is essential to recovery and community integration. In supported housing, service agencies and housing agencies are legally and functionally separated. Services are community based and there is no live-in staff. There is however, 24 h a day, 7 days a week crisis services. Compared to ‘supportive’ housing, ‘supported’ housing is a more independent and permanent housing model. It is based on empowerment, community integration and tenant identity [15, 17, 23]. Supported housing also offers tenants the opportunity to reside in the mainstream housing market by offering financial and social support that will enable them to do so [17]. In both ‘supportive’ and ‘supported’ housing, however, there is a focus on recovery and community integration instead of pathology [3, 17].

The current study falls within the domain of supportive housing that seeks to provide housing stability [3, 5, 17, 18] which is linked to community integration [3, 6, 8, 17], including the adoption of social roles [3, 6, 17], reconnecting with family, securing and maintaining employment [3, 17]. To achieve effective community integration for persons with mental illness, the Federal Government of Canada implemented a supportive housing strategy in 2018. This program recognized persons with mental illness including those with addiction issues as a particularly vulnerable population, saying that transitional and supportive housing must be inclusive [11].

The present study examined the Homes for Special Care (HSC) to CHO (the Community Homes for Opportunity). Over the years, the HSC offered long-term permanent supportive housing in group homes for people discharged from Provincial Psychiatric Hospitals. HSC was managed through the hospitals’ former psychiatric system with 24-hour availability of staff, meals, supervision, and assistance with activities of daily living. Although the HSC provided aspects of supportive housing, it was criticized for being more custodial, and not offering autonomy and tenant choice which are principles of recovery. Basic needs such as clothes and toiletries were provided with little choice. Receiving services was conditional on staying with HSC. For this reason, in 2018, the MOH, St. Joseph’s Health Care (London, Ontario), and community partners started the modernization process of HSC to Community Homes for Opportunity (CHO). CHO gives priority to individuals with serious mental illness who are homeless or those at risk of homelessness, and individuals in hospitals who are not able to care for themselves. The goal is to integrate the CHO program into the mental health and addiction community services and the supportive housing sector [21].

One of the foundations of CHO is to give voice to the tenant in terms of choice and decision-making, including choosing where to live and signing their lease (which never happened under the HSC) in relation to where they would want to live. CHO strives to promote tenants’ independence as much as possible. The CHO is person-centered, and recovery-based meaning tenants are empowered to be active participants in their goal setting and planning and the making of individual choices. CHO focuses on the unique needs of tenants, autonomy, and their integration into the community. To enable this, tenants are provided with increased financial support and encouraged to work on personal growth and life skills through a variety of activities and programs such as learning skills (i.e., furthering education, financial literacy, catering, and personal hygiene), managing their resources, taking part in leisure activities of their choice within the home and in the community. Tenants are also paired with a community social worker. Services are provided both in home and in the community to support their physical and mental health [21].

The overall purpose of the current study was therefore to evaluate the modernization of HSC to CHO in a specific catchment area overseen by St. Joseph’s Health Care London, Ontario, Canada. The ‘evaluation’ comprised examining the nature and experience of tenants in response to the modernization, and to address existing policies and practices that could interfere or promote the modernization. In the end, findings of the CHO from tenants’ perspectives were assessed and a review of the modernization process has since been ongoing.

## Method

### Design

This qualitative focused ethnographic study was part of a larger mixed-methods research project conducted to evaluate the ‘Phase One Homes for Special Care in Southwestern Ontario, Canada. This evaluation examined the nature and experience of tenants of the CHO program (formerly called Homes for Special Care, HSC) in response to the changes being made. Changes were made to modernize, address the current policies and practices to foster or interfere with those changes and provide recommendations to improve the program. The research looked at the outcomes of CHO clients as well as a review of the process to modernize the HSC program in Southwestern Ontario. The focused ethnography design was deemed to be a methodology fit for examining the nature and experience of tenants of the CHO program in response to changes being implemented since this study design, in contrast to classical ethnography, facilitated a timely investigation of health problems within a relatively short period [9, 10, 13, 14, 25] without the researchers necessarily having to be on the field for a prolonged period. A focused ethnography methodology allowed the involvement of persons with experiences, tenants benefiting from the CHO program, to identify relevant insights concerning policies and practices that foster or interfere with clinical intervention [4, 9, 10, 26], which in this case were changes being implemented, under the modernized program.

### Sample and setting

Twenty-eight homes serving 368 tenants in Southwestern Ontario were involved in the evaluation of the CHO program. The 28 homes were the first in the Ontario province to adopt the modernized program that was operated through St. Joseph’s Health Care London. We must emphasize that prior to recruiting the participants, we had had prior connections/interactions with some of these participants due to previous research that we conducted in the homes. Therefore, we already had both formal and informal relationships with some of these participants which aided our recruitment process. After explaining the purpose of the study to them, 188 tenants who consented to take part in the study were enrolled. Over the study period, the 188 participants took part in 28 focus groups, i.e., 10 tenant baseline focus groups in 10 homes (n = 61); nine [9] tenant focus groups in nine homes (n = 60), and nine [9] tenant focus groups in nine homes (n = 67) participants.

### Data collection

The study was approved by the Western University Health Sciences Research Ethics Board. Posters announcing the research study were distributed in the CHO homes for participant recruitment. Focus groups were conducted with CHO tenants to identify issues, solutions, and recommendations for improvement. We used focus group discussion as a qualitative approach to gain an in-depth understanding of the tenants’ experience of social change with the modernization process. Each participant was given a unique code (starting from P1 to P188) to help the researchers to identify participants. Before the commencement of data collection, the researchers reiterated the objectives of the study, after which they obtained informed consent from each participant before starting the focus groups. Data were collected using focus groups which took an average of 60 min each. Focus groups were audio-recorded and then transcribed verbatim by two members of the research team. Note-takers gathered information about group dynamics, context, and non-verbal information, which were integrated into the transcribed data to augment research findings. These notes were inculcated into the description/interpretation of the findings but not into the quotes because as an evaluation of tenants’ experiences, we wanted to project the views/voices of the participants (unadulterated), hence we presented their voices as verbatim quotes. The focus groups were conducted at three time points, i.e., at pre-implementation/Baseline/Time I – spring 2018; Transition/Time II – fall 2018, and Final/Time III – winter 2019. This helped the researchers to remain within the community for sustained periods to understand and to assess the housing situation of the tenants. The baseline data served as a starting point for evaluating the CHO program.

### Data analysis procedure

An ethnographic qualitative analysis based on Leininger [14] was applied to examine focus group data from participants. We used an inductive process to understand the transcribed focus group data attending to what we learned from the data to avoid any preconceived ideas concerning the topic under study. The inductive approach also helped the researchers to derive findings in the context of the evaluation questions. Two graduate research assistants analyzed the data. They first listened to the audio tapes together to acquaint themselves with the raw data after which each of the assistants read through focus group transcripts separately, to identify preliminary codes based on distinct descriptors of ‘what worked well, what did not work well, and suggestions for improvement’. Each of the researchers applied this technique in analyzing data from each site. Data were first coded with descriptive labels. The identified codes were then categorized alongside their respective exemplar quotes into subthemes based on their meaning in relation to the context of participants’ views. The categories were developed into subthemes based on identified meaning.

The focus group data involving time – I, II, and III were analyzed separately. Haven identified all subthemes from each site, the researchers then aggregated them based on similarities and differences between the findings in relation to each time point. Since the baseline data served as a starting point for evaluating the CHO program, baseline findings were not added to the results in this paper. In the end, the emergent themes from time –II and III focus groups were further aggregated and grouped based on commonalities in relation to facilitators, benefits, barriers, and challenges throughout the CHO program’s implementation, as well as solutions, and recommendations for improvement. To ensure the credibility and trustworthiness of the study findings, all members of the study team were given copies of the results to appraise and to make inputs in the form of comments. All comments and observations from the co-researchers were then integrated into the final research document.

## Results

Study participants were between 23 and 79 years, and 42% were females. 74% of participants were single, while 19% were separated or divorced. Only 3% were married or in a common-law relationship, and 4% were widowed. Most participants (67%) received income from the Ontario Disability Support Program (ODSP). About 95% of the sample had at least one psychiatric diagnosis. The common diagnoses among participants include schizophrenia, mood disorder, anxiety disorder, and substance-related disorder. Most of the participants identified themselves as Caucasian (79%). The remainder identified as Aboriginal (4%), a visible minority (7%), or other (9%). Data analysis of tenants’ perspectives about the CHO implementation program revealed four major themes. These include: [1] General impression about CHO Program, [2] perceived benefits of CHO, [3] perceived barriers while implementing CHO, and [4] strategies for improving CHO.

**Fig. 1:**
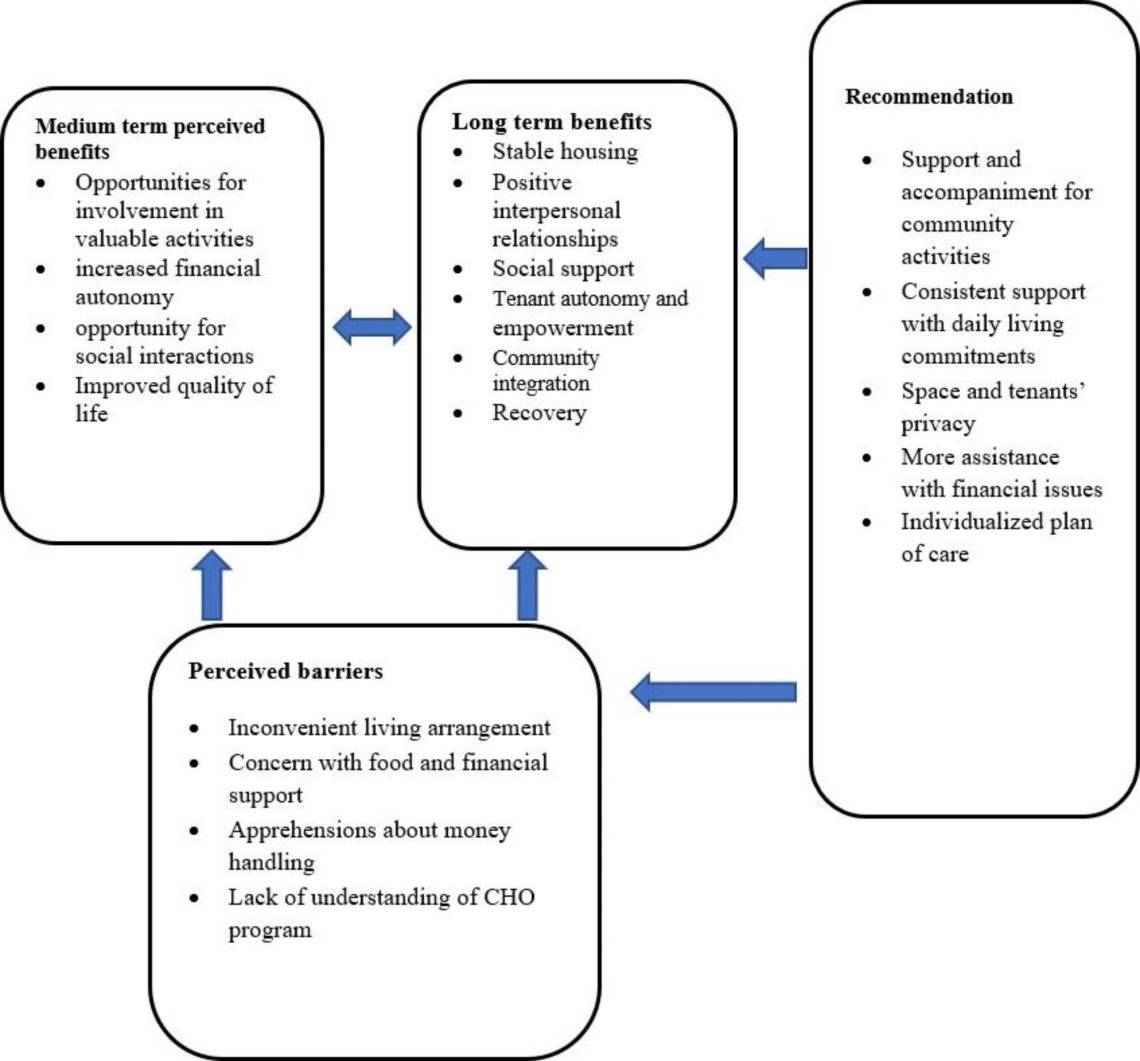
A CHO framework depicting perceived barriers and long to medium term benefits for tenants of the modernized program

Despite the barriers that appear to affect the successful implementation of the CHO program, the findings demonstrate that tenants experienced both medium and long-term benefits as shown in Fig. 1 below. In this framework, the proposed strategies to improve CHO if implemented would enable long-term benefits for the tenants. Alternatively, a lack of effective implementation of the tenants’ suggestions will likely create barriers for the CHO program.

### General impression about CHO program

In relation to impressions about what had gone well with the CHO, the tenants had positive feelings about the changes brought about by the modernized program such as staff and other services. Some tenants spoke well of the Agency staff; that the staff were great and supportive. Tenants’ appreciation of services, and satisfaction with the program related support, fostered autonomy and positive interpersonal relationships in the homes.

#### Tenants’ appreciation of services

The tenants were happy with the CHO program. They were thankful and showed admiration towards staff for the immense support given them.

A participant said:

“I feel like the staff is very professional, they are very friendly, very approachable, the food’s good, I mean, it’s been a good experience, family is at peace knowing I’m not, out there. This program has been a blessing for me.” [P20].

Another participant added:

“Yup, I know what you mean now. This place they feed you good, they look after you good. They treat you very well. I think everybody appreciates the CMHA [agents providing services] a lot more than they did the other round [referring to the homes for special care]” [P61].

#### Satisfaction with the program-related support

The tenants expressed great satisfaction with the support they were getting under the CHO. They showed delight about the show of empathy from the staff. The tenants also expressed gratitude for the financial package that came with the modernized program (CHO); that, the immense support was going to assist most of them to prepare adequately towards independent living in the future.

One of the participants revealed:

“It’s always clean. The staff [home staff] is good at cleaning and cooking and making sure that you are making your appointments on time, making sure you take your meds. I would like to add, this home; this place feels like home to me. Nice people, nice staff, nice food, nice everything. I don’t want that to change.” [P25].

This participant also added:

“The funding to cover everything, is a great idea because there are some people that would want to get their own independent living apartment, and they might need household furniture, and things that they want for their little apartments. Like the senior apartment, everything they need for their apartment when they get out on their own”. [P156]

#### CHO fostering autonomy

Tenants revealed that the CHO nurtured independence among them, thus, enhancing their self- confidence and competences in the management of their own resources.

A participant said:

“I think it’s a better service, it’s a good idea [referring to CHO]. I like this idea because everybody has extra money to go out. They wanna go out on a social event, or a ride somewhere. They have the money to go to the store to buy ice cream or buy clothes. I think it’s really good.” [P11].

Another participant said:

“I talked to my trustee and told her I can manage my own finances through my ODSP [the Ontario Disability Support Program] cheques that I have been getting. I just got onto CHO. I will be responsible for paying my bills, my food, everything.” [P117].

#### Fostering positive interpersonal relationships

Most tenants gave a good impression about their fellow tenants in the homes. The researchers observed that relationships between tenants were very cordial and that there were no safety issues, including unwarranted suspicions or fear of being hurt by another person. This positive attitude from the tenants has been influenced by the CHO implementation process and the fact that the staff also interacted with family members.

A participant said:

“With people that live in this home all have a lot in common. You don’t have to worry about anyone stealing or hurting you. You can just be yourself, that’s the main thing that I think is good about this place. We live like one happy family, no arguments no fighting, which is good.” [P15].

Another participant added:

“The CHO program has been there for me. Also, she [the social worker] talks to my mother too, so that way the communication lines are open and she can discuss things with my mom. I found a really big change. She cares a lot more…” [P26].

### Perceived benefits of CHO

Tenants benefited from the new CHO program in various ways. For instance, the participants expressed excitement about the program, and that it offered them unique opportunities and support for involvement in several activities. The tenants also revealed that the program enhanced their quality of life and well-being. As captured in the field notes, participants’ social interaction was cordial; they showed collegiality and supported each other. The study participants also demonstrated autonomy in terms of personal and financial independence by making their food choices, including the type of clothing they wanted to buy.

#### Opportunities for involvement in valuable activities

The autonomy that is associated with the CHO allows tenants to engage in various forms of activities they felt were important to them. They perceived such activities as forms of extra-support. For instance, this tenant revealed how the CHO program offered him the opportunity to further his education.

“I’m going back to the school for my university degree. I’m taking an IT course there…” [P85].

This participant also added:

“I would save to buy a cell phone and a textbook that I require for a course that I’m taking over the internet and I was able to pay for that with the extra money of which I would’ve never been able to do.” [P126].

#### Involvement in social interactions

CHO offers opportunities to all tenants to be able to take part in leisure activities of their choice within the home environment and in the broader community. In the end the interactions that tenants have with each other go a long way to improve group support, cohesion and sense of entitativity, where they view themselves as one group with a common background.

This participant indicated:

“We try and make sure we don’t hurt each other’s feelings…If someone’s doing something wrong, I will speak. I’ll say you’re not supposed to be doing that. You could try not doing something else…you don’t get lonely in the group homes. It’s best to be with somebody in a room.” [P13].

One of the participants also said:

“They have different things on different days. Like Tuesdays there are men’s and women’s club that they do museums and stuff like that but you have to go to that program to get these things.” [P36].

#### Improved quality of life

Increase in the amount of money per tenant has raised the purchasing power of every individual towards being able to afford the cost of necessary commodities, culminating into an improved lifestyle among tenants. For instance, getting more money was an asset that aided frequent clothing shopping, and more options for different varieties of goods for the participants. In another vein, the fact that tenants had a say in food choices empowered them while enhancing their nutritional status simultaneously.

This participant said:

“I’m happy, I’m happy! Home, food, and everything! I love it here! I like it, yeah…we go by the health and food guide of Canada, so when they’re making meals we look at the food guide for sandwiches where that’s not good then you don’t, they don’t make that food.” [P118].

Another one added:

“Long time ago, I wouldn’t appreciate it [money], I just bought a lot of booze. [currently], I’d buy grocery, I’d buy things I need, like make up, hair dye, and earrings, and, and things I really need, like underwear, and bras, dresses, and high heels.” [P56].

#### Financial empowerment

The CHO enabled the tenants in various life domains including assisting them acquire the day-to-day financial management skills that made them take good decisions towards the healthy spending of the money given them, including enhanced opportunity for savings. The increase in financial support is a watershed moment for the tenants; a crucial moment that continues to turn lives around for the better. The participants of CHO revealed how the increase in financial support gave them the opportunity to start saving money for the future so they could live independent lives.

A participant expressed this view:

“I’m pretty happy now, like I mean I don’t go into the community a lot but like I go for coffees, I go to the stores you know so…I’m usually busy.” [P76].

Another participant added:

“I’ve got a savings account…I mean thinking about that I think if I put a little money away each time, I’ll be able to manage it well…I get very few clothes because I save them for years and keep them. So I don’t spend much on clothes so that will benefit me there because I don’t spend much anyways.” [P101].

### Perceive barriers while implementing CHO

In terms of what did not go well with the CHO experiences of tenants, researchers documented issues related to inconvenient living arrangements, concerns with food and financial support, concerns about money handling, and the lack of understanding of the CHO program, were key obstacles that the tenants faced in the homes.

#### Inconvenient living arrangement

Tenants expressed concerns about limited amenities culminating in a lack of privacy and an overcrowded environment. They felt this situation threatened their comfort, confidentiality, and individuality. It is notable however that this issue of historical ‘inconvenient living arrangement’ in terms of physical structures has existed since the HSC and continues to exist with the CHO program since it takes place within the same buildings. Some of the tenants’ sentiments include the following.

“Maybe if they had a little fridge where we can put our own snacks, because we are not able to keep our food anywhere. We can’t have it in room.” [P16].

“I find this place very crowded. And the television room, there’s a lot of people here, using the television… It would be nice if we have our own rooms. But that’s impossible because there’s like 30 people.” [P161].

“The only trouble is there could be more than one person in your room. That’s kind of private but you got to share. I think they should have a room for each person in the group homes. That’s what the problem is.” [P126].

#### Apprehensions about financial support and money handling

Some tenants reflected and raised concerns that other tenants would not use their own money appropriately (in terms of the purpose for which the money was given, i.e., as monthly spending money). Tenants also raised issues with the financial assistance they were receiving. For instance, they described the amount of money given to them for living as insufficient despite the increase (from $150 to $500 a month). Some of the tenants’ concerns have been presented as follows.

“It [money] just is, you know, it’s barely; you can’t live off it at all. By the time I pay my bills I’m broke.” [P42].

Another person said:

“People might not have enough for like emergencies, you might need a pair of shoes and you don’t have any money, so what’s gonna happen to this person who needs shoes?” [P81].

#### Lack of understanding of CHO program

Tenants appeared to have little knowledge about CHO programs despite communications from the ministry to the homeowners (private persons who own the houses) and individuals directly. For example, some areas such as transportation and rent payment needed more clarification in terms of the amount of money covered by the program versus the amount of money paid by the tenants.

One of the tenants questioned:

“Somewhat confusing at times [referring to CHO] not knowing like say I’m needing to go to the hospital, and how do I get home? Do you guys cover [transport]?… that need to be worked out in my opinion.” [P126].

### Proposed strategies to improve CHO

Overall, all tenants believed that the modernization of the HSC program to CHO was an improvement. For instance, the tenants were satisfied with the increased financial assistance and one-on-one support from the Agency staff. However, the tenants identified some areas that needed improvement. They called for more support and accompaniment for community activities, consistent support with daily living commitments, more assistance with financial issues, ensuring individualized plans of care, and the need to address historical concerns with space and privacy in the homes.

#### Support and accompaniment for community activities

Even though the CHO seeks to promote autonomy among tenants, the tenants still requested continued assistance from the attending Agency staff and homeowners during external activities such as going to the shop, the bank, and other community events.

One of the participants advocated:

“More activities within the community offered for people. Y (YM/WCA gym) passes should be covered for young and old… for people that can’t afford it, they should be able to get their Y passes and stuff…Like take us out to go shopping, clothes shopping, stuff like that…They got a quarry here, maybe sometime they’ll be able to take us to the quarry to do something. It’s a nice swimming spot.” [P51].

Another person also requested; “I’d like somebody. I need someone to take me to go out and do something with. I’d like someone to go to the bank with twice a week. I don’t have anybody, I’d like somebody.” [P117].

#### Consistent support with daily living commitments

Tenants requested that the homes should provide consistent assistance in relation to helping them in handling daily life issues. The tenants felt that maintaining the same staff in the homes was beneficial. Again, some proposed the inclusion of peer supporters (i.e., other people with a history of mental illness) in the sense that they could get extra support from these individuals to enhance their social skills and well-being.

A participant recommended:

“…I would like it if they [Agency staff] could keep constant stay here like, permanent stop, not just for one year or six months… I don’t like it….” [P116].

One of the participants also proposed:

“…getting some sort of peer support, like one-on-one sort of thing, like I have in the past. I had somebody in the hospital, and that meant a lot. I think that’s important a peer support group or system.” [P118].

#### More assistance with financial issues

In as much as the tenants appreciated the enhanced support offered them by the new program, they still asked for more financial assistance as well as some guidance with appropriate financial resource management skills to help them benefit more from the assistance given to them.

This participant said:

“Some of us here aren’t going to be able to do it, have to have assistance budgeting”. [P29]

#### Individualized plan of care

Even though tenants were very excited with the new program (CHO), most of them felt that certain aspects of the old program (Homes for Special Care) be maintained so homeowners and other staff could continue to help with essential services especially to those with special needs.

One of the participants indicated:

“It’s up to us from our own pocket money to buy the bus pass, so I like the old system. [They] dropped off a bus pass for each month. I think that was a good system…I guess the old way is pretty good, it was all organized. Everything was organized and we got help with everything.” [P12].

“That was good! Bus fare, transportation to get to doctors’ appointment, and haircut, you know emergency stuff, should be left alone.” [P171].

Another participant gave other reasons for an individualized support:

“Well, essential services such as bus pass, is essential for some people, transportation of course, the haircut, toiletries. Some people either can’t get to the Dollarama because of mobility, or maybe the time of year or the weather…there may be some in the system that may not be able to budget for the essential services that they are provided by whatever income they get…” [P141].

## Discussion

The study findings suggest that the transition of HSC to CHO supports activities that empower tenants towards personal growth and development. Major findings from the study indicates that even though the tenants had a good impression about the CHO program stemming from the benefits that accrued to them, some still encountered problems and provided suggestions to further improve the existing program. For instance, the tenant participants called for more support and accompaniment for their community activities, including consistent support with daily living commitments, as well as more assistance with financial issues, ensuring individualized plan of care and addressing historical concerns with space and privacy in the homes.

In another vein, tenants expressed positive feelings about the changes brought about by the modernized program. Study participants were largely satisfied with the support they were getting from the staff in relation to the program-related services. The tenants were satisfied with the level of empathy the staff displayed while assisting them in fostering positive interpersonal relationship. The tenants also expressed gratitude for the enhanced financial package that came with the modernized program. They believed the immense support was helping most of them to prepare adequately towards independent living in the future. This is in line with the recovery-oriented perspective of the CHO program, as it enables individuals affected by mental illness to live independent, satisfying, hopeful, and productive lives despite the limitations caused by their illness [1, 12].

One of the key goals of supportive housing programs such as the CHO, is fostering autonomy and independent community living. Tenants’ independence facilitates their self-confidence and competencies in the management of their own resources. The supportive housing model also foster recovery with more emphasis on recovery of the individual. The findings indicate that CHO fosters recovery among tenants using both informal and formal supports or resources. The CHO program also recognizes all tenants as experts in what they need to facilitate their own recovery, and part of a circle of shared decision making that fosters autonomy in the end [7]. This finding also resonates with Stanhope and colleagues [20] observation that for one to achieve a healthy interaction that enhances autonomy, decisions should be based on the care receiver’s experiences and the provider’s expertise working together.

The study findings demonstrate that the CHO program offers tenants opportunities and support for involvement in various activities in-house and in the community. Tenants described feelings that their quality of life and well-being had been enhanced through participating in the program, and that their social interaction and support for each other had also improved. Most tenants demonstrated autonomy in terms of personal and financial independence. The autonomy connected with the CHO program gives tenants the opportunity to engage in various forms of activities that are meaningful to them. Such activities act as extra-support to reduce boredom, enhanced group cohesion and sense of entitativity, where they view themselves as one group with a common background. The ‘good feeling’ outcome associated with both in-house and community programs ultimately contributes to the tenants’ sense of worth and belonging in the community. These unique outcomes of the program evaluation underscore the importance of stable supportive housing not only in Canada, but the world at large. For instance, Chan [3], Doroud, and colleagues [6], and the Mental Health Commission of Canada [15] believe that stable supportive housing is an essential part of the pathways to community integration that offers social connection, feeling of belonging and participation in one’s community especially among persons with severe mental illness.

The study findings again reveal that the CHO program offers tenants the opportunity to become financially autonomous in terms of deciding on when and how to spend their money and other resources available to them. The enhanced financial support for tenants does not only improve their quality of life, but also helps to raise their purchasing power, decision making abilities, sense of responsibility and accountability towards the healthy spending of the resources entrusted to them. Overall, we assert that the CHO program aids in the adoption of unique social roles for community integration just as Chan, [3], Doroud, and colleagues [6], Farkas, and Coe [8] and Nelson and company [17] have argued elsewhere.

Despite the unique benefits that implementing the CHO program offered to tenants, the study findings reveal some difficulties that tenants face in the homes. For instance, there were issues related to a lack of understanding of the CHO program. Participants also had concerns with their food menu, the financial support, and how others were handling the monies given them. Inasmuch as tenants were benefiting from the CHO program, they also expressed concerns about limited amenities in the homes that invariably led to lack of privacy and overcrowded environment. This situation may likely threaten tenants’ comfort, as well as the confidentiality that each one needs as unique individuals and to facilitate independence and recovery. This finding has implications for future planning and choice of homes for the supportive housing program. A needs assessment should be considered especially during the screening period so that tenants could be appropriately matched with homes that meet their unique needs. This is in line with the assertion made by Montgomery and colleagues [16] that individual supports ought to be driven by the person’s needs for well-being.

Tenants also raised issues with food menu in some of the homes. This underscores a need for good communication, targeted education towards effective resource management and involvement of tenants during the planning and execution of all activities in the homes. Considering everyone’s unique decision-making ability especially when such decision outcomes have a direct bearing on all in the home [7] is vital for cohesion and positive experience of the CHO program.

### Implications of the study

Supportive housing programs such as the CHO program can provide stable housing. Despite the challenges, study findings revealed that the modernization of the HSC program to CHO was beneficial for personal growth and development toward one’s social integration. This finding points to the need for implementation of the CHO program across Ontario in short to medium term, and for encouraging its uptake in other provinces.

Tenant perspectives on the implication of the CHO program provide key insights for homeowners and their staff, as well as key stakeholders such as policymakers, and Community Mental Health Agencies. The implications range from strategic investments to operational changes. There is a need for strategic planning that is more tailored to needs assessment and individualized services that are apt for tenants’ community integration in the long run. Research concerning tenants’ views on supportive housing for individuals with mental health and addiction problems is limited. Thus, this study contributes to knowledge advancement, with far reaching implications for academia, family members involvement, and improved social advocacy for the marginalized.

### Limitations

This study has strengths and limitations. A highlight of the study has been that tenant beneficiaries of the evaluated model were actively involved in all stages of the study. The study was not only undertaken in the research participants’ (tenants’) settings but also provided them with an opportunity to reflect on their experiences of both the previous model (HSC) and the modernized program (CHO). As research, the study approach facilitated tenants’ participation that generated findings that are relevant and tenant-specific; helping to uncover the actual nature of the program as well as enablers and barriers to the effective implementation of changes under investigation. However, the findings may only be transferrable to contexts and settings that are similar to those of this study. Personal experiences such as those expressed by our study participants are time and context dependant. Therefore, the tenants’ views about the CHO program may evolve over time. As such, there would be a need for continuous evaluation of the program for improvement and sustainability.

## Conclusion

Supportive housing models such as the CHO constitute an effective pathway to ending chronic homelessness for people living with mental illness or addictions. Stable housing is a right, and a fundamental need of every human being towards good health and growth [24] making it even more urgent to continue with the CHO, an innovative, recovery-based housing intervention for individuals living with serious mental illness. It is expected that a more effective and expanded CHO will lead to tenant empowerment or recovery and successful social integration of persons with mental illness and those with addiction.

## Data Availability

Datasets generate and/or analyzed during the current study are not publicly available due to participant privacy but are available from the corresponding author on reasonable request.

## List of abbreviations

CHO: Community Homes for Opportunity
HSC: Homes for Special Care
